# Supplementary material for: Electronic Structures of Radical-Pair-Forming Cofactors in a Heliobacterial Reaction Center
Source: Molecules. 2024 Feb 27;29(5):1021. doi: 10.3390/molecules29051021 (PMC10934547; doi:10.3390/molecules29051021)
Supplement: Supplementary file 1 [file molecules-29-01021-s001.zip › molecules-2871399-supplementary.pdf]

Electronic Structures of Radical-Pair-Forming Cofactors in a Heliobacterial Reaction Center

Yunmi Kim [1], A. Alia [2, 3], Patrick Kurle-Tucholski [1], Christian Wiebeler [1,4] and Jörg Matysik [1,*]

[1] Institut für Analytische Chemie, Universität Leipzig, Linnéstraße 3, D-04103 Leipzig, Germany; yunmi.kim@uni-leipzig.de (Y.K.); patrick.kurle@uni-leipzig.de (P.K.-T.); christian.wiebeler@uni-a.de (C.W.)

[2] Leiden Institute of Chemistry, Leiden University, Einsteinweg 55, 2301 RA Leiden, The Netherlands, alia.aliamatysik@medizin.uni-leipzig.de (A.A.)

[3] Institut für Medizinische Physik und Biophysik, Universität Leipzig, Härtelstr. 16-18, D-04107 Leipzig, Germany

[4] Institut für Physik, Universität Augsburg, Universitätsstraße 1, D-86159 Augsburg, Germany

* Correspondence: joerg.matysik@uni-leipzig.de; Tel.: +49-341-9736112 (J.M.)

Supporting Information Available

Figure S1. Comparison of ^{13}C photo-CIDNP MAS NMR spectra from ^{13}C natural abundant intact cells and isolated membrane fragments

Figure S2. Schematic structures of chlorophyll

Figure S3. 2D ^{13}C–^{13}C photo-CIDNP DARR MAS NMR spectra of [3,4,5-^{13}C]-ALA with a mixing time of 2.5 ms obtained at 4.7 T.

Table S1. ^{13}C-^{13}C correlation pairs (cross peaks) from electron donor and acceptor

Figure S4. Correlation between the experimentally obtained ^{13}C chemical shifts and the calculated ^{13}C magnetic shieldings.

Figure S5. Correlation between experimental photo-CIDNP intensities and DFT calculations.

Table S2. Relative ^{13}C photo-CIDNP intensities of the electron donor, BChl *g*' and calculated spin density distribution of donor triplet state.

Table S3. Relative ^{13}C photo-CIDNP intensities and calculated principal hyperfine coupling tensors of electron acceptor, 8^1-OH Chl a_F

Figure S6. Mapping the local electron spin densities of ^{13}C and ^{15}N in both the donor triplet state and radical anion state within the HbRC.

Table S4. ^{13}C chemical shifts of aromatic region of active cofactors (donor and acceptor) observed by photo-CIDNP MAS NMR experiment of HbRC, the RCs of *R. sphaeroides* WT and PSII (spinach).

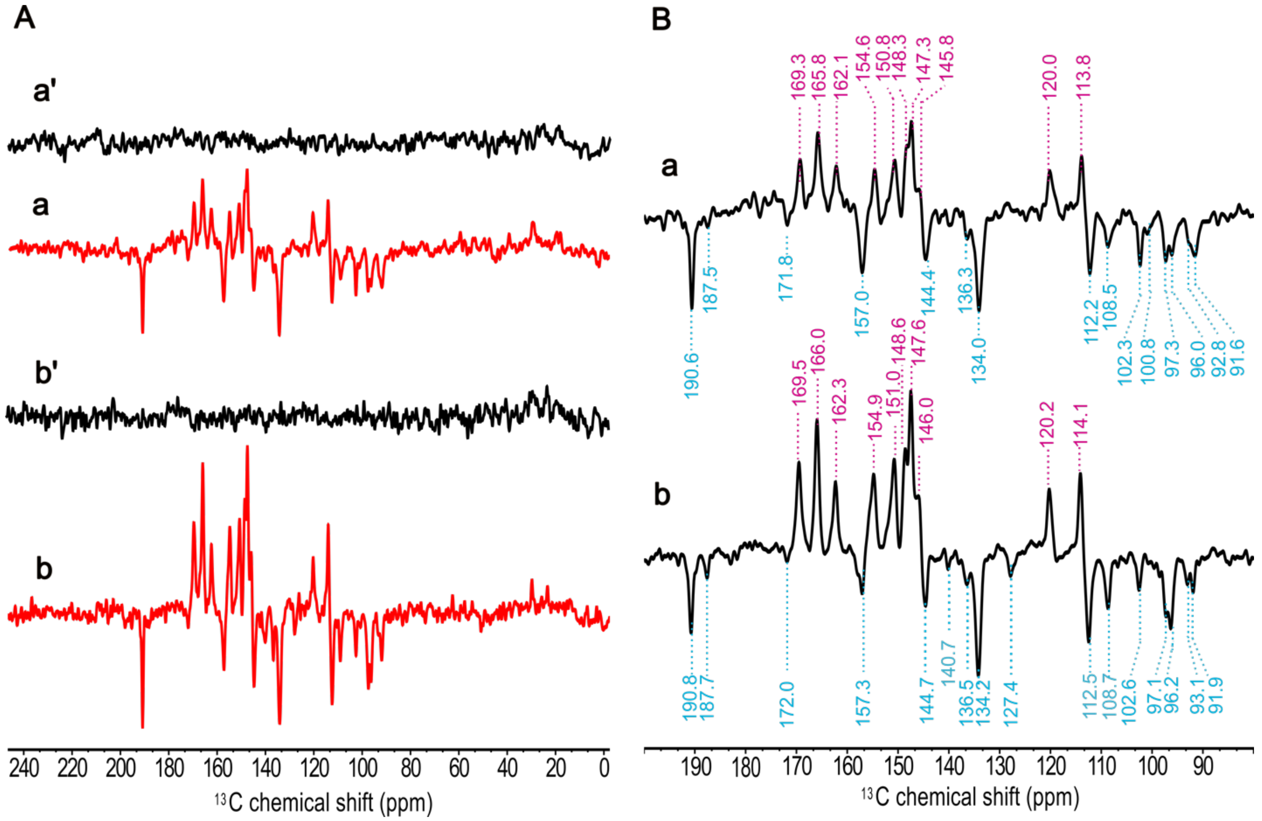

Figure S1. Comparison of ^{13}C photo-CIDNP MAS NMR spectra from natural abundant intact cells (**a**) and isolated membrane fragments (**b**) from *Hb. mobilis* are shown in red color. The MAS NMR spectra obtained in the dark of intact cells (**a'**) and isolated membrane fragments (**b'**) are shown in black. All the spectra have been collected for 10 h under illumination with 488 nm at magnetic field of 4.7 T with a cycle delay of 4 s for 10 h. A MAS frequency of 8 kHz was employed for the experiments conducted at 235 K. Expanded light induced spectra of intact cells (**a**) and membrane fragments (**b**) in **(B)**.

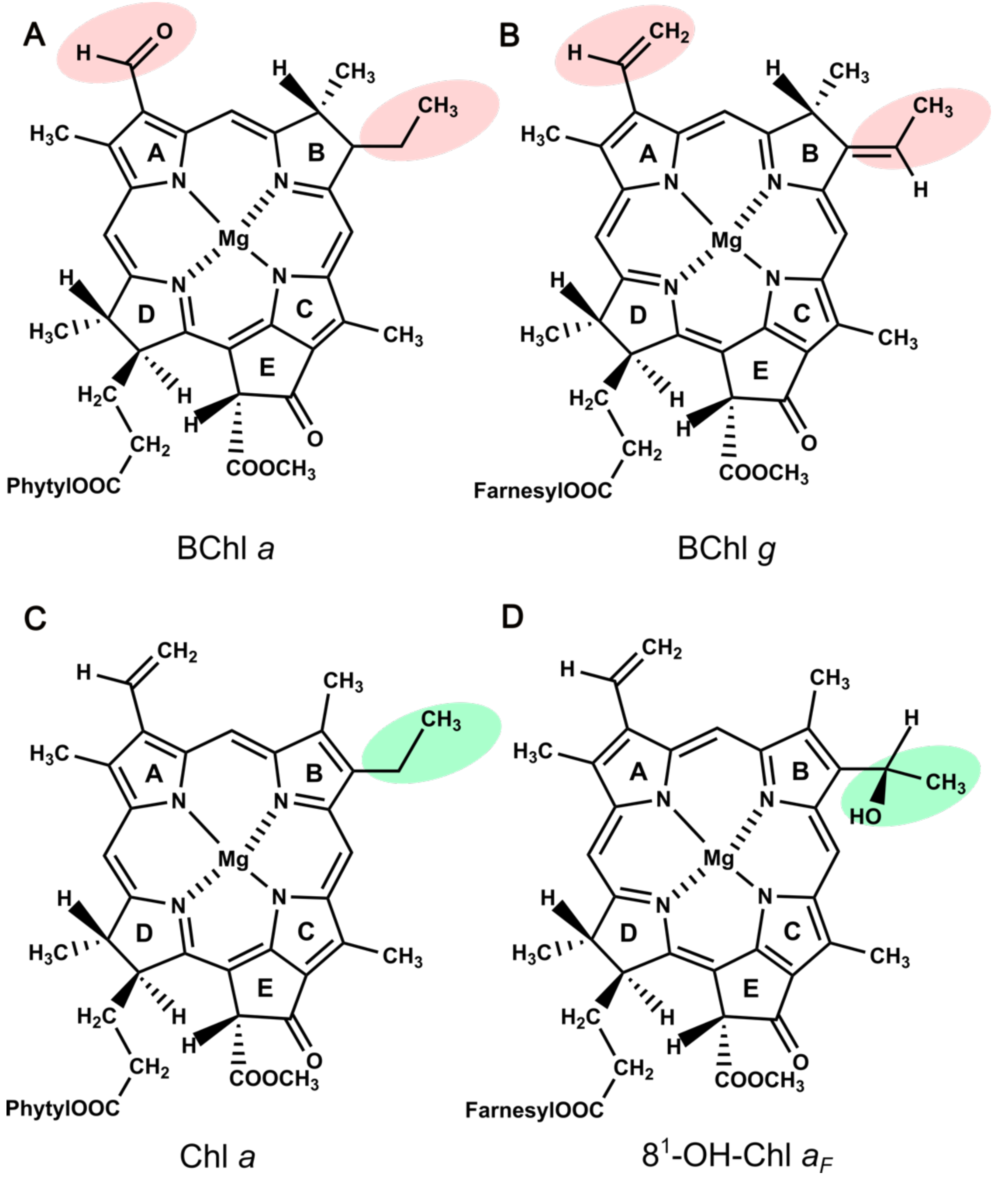

Figure S2. Schematic structures of Chlorophyll. BChl *a* (**A**), BChl *g* (**B**), Chl *a* (**C**), 8^1-OH-Chl a_F (**D**), [1,2]. The modifications in rings A and B between BChl *g* and BChl *a* are marked in red, while the divergence within ring B between 8^1-OH-Chl a_F and Chl *a* is indicated in green.

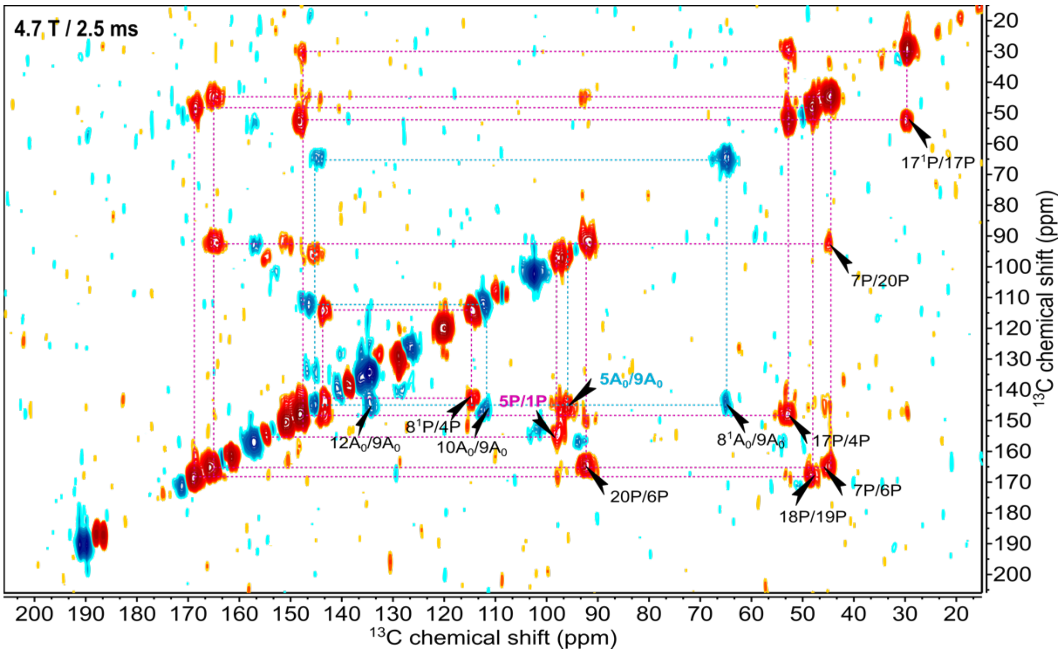

Figure S3. 2D ^{13}C–^{13}C photo-CIDNP DARR MAS NMR spectra of [3,4,5-^{13}C]-ALA labelled membrane fragments with a spin-diffusion mixing time of 2.5 ms, collected with a MAS frequency of 8 kHz at 235 K under the continuous illumination with 488 nm measured at 4.7 T. Positive contour are indicated in red, negative contour are marked in blue in the spectra.

Table S1. ^{13}C-^{13}C correlation pairs (cross peaks) from electron donor and acceptor observed in 2D ^{13}C-^{13}C DARR spectra with a mixing time of 50 ms at 4.7 T and 9.4 T.

	electron donor, P, BChl *g'*		electron acceptor, A_0, 8^1-OH-Chl a_F	
No.	Correlation pair	Distance, (Å)	Correlation pair	Distance, (Å)
1	4P/17^1P	6.2 Å (inter)<br>7.3 Å (intra)	$1A_0/2A_0$	1.4 Å
2	4P/17P	6.1 Å (inter)<br>6.5 Å (intra)	$3A_0/2A_0$	1.5 Å
3	5P/9P	3.6 Å	$3A_0/13A_0$	8.4Å
4	6P/7P	1.5 Å	$6A_0/10A_0$	3.5 Å
5	6P/20P	5.5 Å	$6A_0/11A_0$	4.2 Å
6	7P/4P	3.8 Å	$8^1A_0/9A_0$	2.5 Å
7	7P/20P	6.5Å (inter)<br>6.9Å (intra)	$10A_0/9A_0$	1.5 Å
8	8P/8^1P	1.5 Å	$10A_0/13^1A_0$	4.9 Å
9	8P/4P	4.6 Å	$10A_0/11A_0$	1.5 Å
10	13P/16P	3.7 Å	$12A_0/9A_0$	3.9 Å
11	13^1P/13P	1.5 Å	$12A_0/10A_0$	2.6 Å
12	13^1P/14P	2.3 Å	$12A_0/11A_0$	1.6 Å
13	16P/17P	1.5 Å	$13A_0/13^1A_0$	1.5 Å
14	17P/17^1P	1.5 Å	$13A_0/14A_0$	1.4 Å
15	17^1P/16P	2.5 Å	$13^1A_0/10A_0$	3.5 Å
16	18P/19P	1.5 Å	$13^1A_0/12A_0$	2.8 Å
17	20P/17P	3.8 Å	$13^1A_0/13A_0$	1.5 Å
18	8^1P/4P	5.5Å (inter)<br>6.1Å (intra)	$13^1A_0/14A_0$	2.3 Å
19			$13^1A_0/17^1A_0$	4.7 Å
20			$13^1A_0/18A_0$	5.9 Å
21			$14A_0/12A_0$	3.5 Å
22			$15A_0/6A_0$	6.3 Å
23			$15A_0/14A_0$	1.4 Å
24			$17A_0/1A_0$	4.8 Å
25			$17^1A_0/1A_0$	5.7 Å
26			$17^1A_0/17A_0$	1.5 Å
27			$18A_0/1A_0$	3.9 Å
28			$18A_0/17^1A_0$	2.5 Å
29			$18A_0/19A_0$	1.5 Å
30			$20A_0/1A_0$	1.5 Å

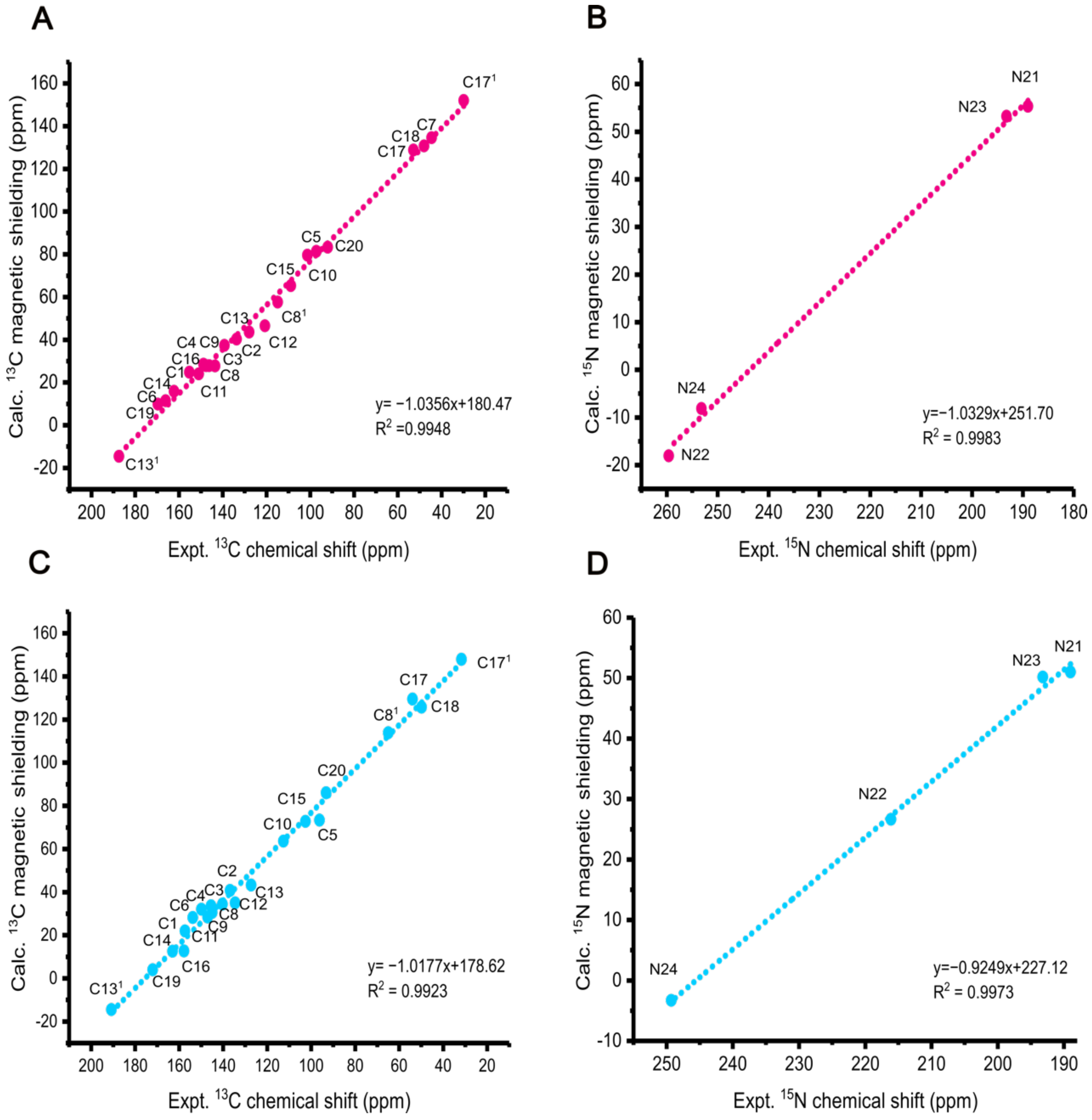

Figure S4. Correlation between the experimentally obtained chemical shifts and the calculated magnetic shieldings for electron donor, BChl *g*', and electron acceptor, 8[1]-OH-Chl a_F. ^{13}C of electron donor, BChl *g*' (**A**), ^{15}N of electron donor, BChl *g*' (**B**), ^{13}C of electron acceptor, 8[1]-OH-Chl a_F (**C**), ^{15}N of electron acceptor, 8[1]-OH-Chl a_F (**D**). The dashed line represents a linear fit. With the parameters obtained from that and given as insets, we determined the chemical shifts based on the computed magnetic shieldings.

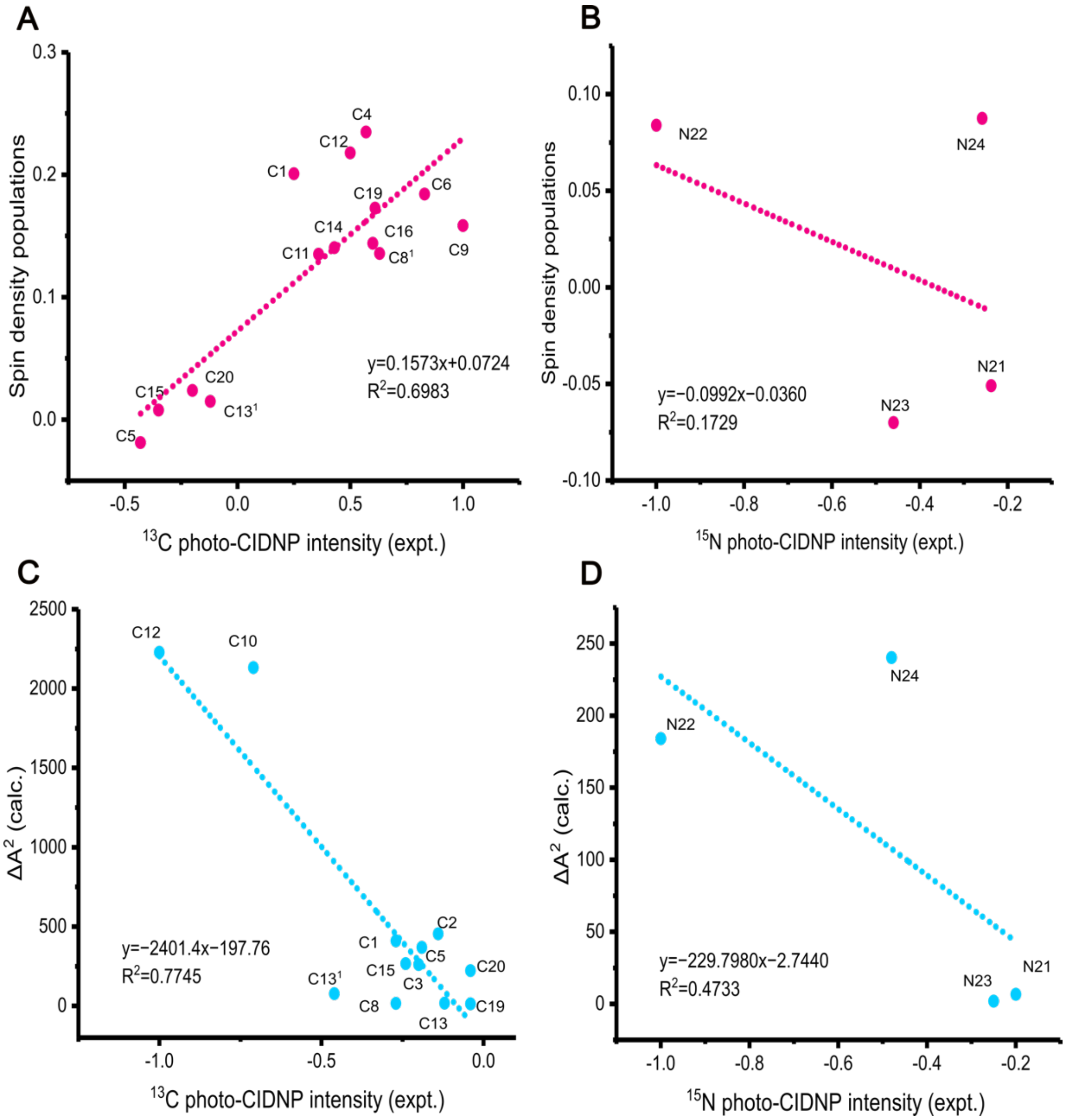

Figure S5. Correlation between calculated spin densities of donor triplet neutral state and experimental ^{13}C photo-CIDNP intensities (**A**) and ^{15}N photo-CIDNP intensities (**B**) of electron donor cofactor, BChl *g*'. Correlation between calculated square of ^{13}C anisotropic hyperfine couplings, ΔA^2 in the radical pair anion state and experimental ^{13}C photo-CIDNP intensities (**C**) and that of ^{15}N photo-CIDNP intensities (**D**) of electron acceptor, 8^1-OH-Chl a_F .

Table S2. Relative ^{13}C and ^{15}N photo-CIDNP intensities of the electron donor, BChl *g*' and spin populations from Mulliken charge analysis based on the DFT calculation for donor triplet state. The relative photo-CIDNP intensities are normalised to the highest enhanced signal.

IUPAC no.	electron donor, BChl *g*'	
	Rel. photo-CIDNP intensities	Spin Populations
N21	−0.46	−0.0701
N22	−1.00	0.0839
N23	−0.24	−0.0509
N24	−0.26	0.0874
$C13^1$	−0.12	0.0148
C19	0.61	0.1726
C6	0.83	0.1841
C14	0.43	0.1403
C1	0.25	0.2009
C16	0.60	0.1440
C4	0.57	0.2348
C9	1.00	0.1584
C11	0.36	0.1350
C8	_	−0.0622
C3	_	0.0460
C2	_	0.0957
C12	0.50	0.2177
C13	_	0.0028
$C3^1$		−0.0268
C10		0.0679
C15	−0.35	0.0077
C5	−0.43	−0.0190
C20	−0.20	0.0237
$C8^1$	0.63	0.1356
C17	_	−0.0069
C18	_	−0.0070
C7	_	−0.0145
$C17^1$	_	0.0080

Table S3. Relative ^{13}C and ^{15}N photo-CIDNP intensities together with principal hyperfine coupling tensor components (A_{xx}, A_{yy}, A_{zz}) and hyperfine anisotropy (ΔA)* from DFT calculations for radical anion state of the electron acceptor, 8^1-OH-Chl a_F. The relative photo-CIDNP intensities are normalised to the highest enhanced signal. $^*\Delta A = A_{zz} - (A_{xx} + A_{yy})/2$

IUPAC no.	electron acceptor, 8^1-OH-Chl a_F				
	Rel. photo- CIDNP intensities	A_{xx}	A_{yy}	A_{zz}	ΔA^*
N21	–0.20	–0.4632	–0.5152	–3.0483	–2.5591
N22	–1.00	–0.5062	–0.8400	12.8887	13.5618
N23	–0.24	–0.1581	–0.2626	–1.5625	–1.3522
N24	–0.48	–0.6647	–0.9289	14.7027	15.4995
$C13^1$	–0.46	–2.4454	–3.4625	5.8249	8.7789
C19	–0.04	0.0382	–5.1689	–6.1449	–3.5796
C6	_	–3.3074	–5.1041	–6.1924	–1.9867
C14	_	1.4480	–2.3033	–2.8259	–2.3983
C1	–0.27	–3.0928	–3.9093	16.7274	20.2285
C16	_	–5.1076	–6.1252	–6.7883	–1.1719
C4	_	–2.3465	–3.0161	11.7731	14.4544
C9	_	–9.0819	–10.5905	–20.9684	–11.1322
C11	_	–10.4550	–11.6159	–12.2899	–1.2545
C8	–0.27	1.4554	1.7801	5.6359	4.0182
C3	–0.20	–3.4671	–3.7998	12.5049	16.1384
C2	–0.14	–4.0087	–4.1804	17.2217	21.3163
C12	–1.00	–0.0013	–0.3822	47.0085	47.2003
C13	–0.12	–1.7366	–7.2541	–8.6588	–4.1635
$C3^1$	_	–3.3931	–3.8980	–6.7492	–3.1037
C10	–0.71	0.5417	0.6769	46.7797	46.1704
C15	–0.24	–1.0938	–1.4273	15.0635	16.3241
C5	–0.19	–2.3459	–2.6928	16.6668	19.1862
C20	–0.04	–3.4657	–4.0764	11.1384	14.9095
$C8^1$	_	–0.4432	–0.6803	–0.7641	–0.2024
C17	_	0.0000	0.0563	–0.3653	–0.3935
C18	_	–0.9027	–1.0728	–1.2477	–0.2600
C7	_	–1.2166	–1.3661	–8.6795	–7.3882
$C17^1$	_	0.8476	1.0792	1.3027	0.3393

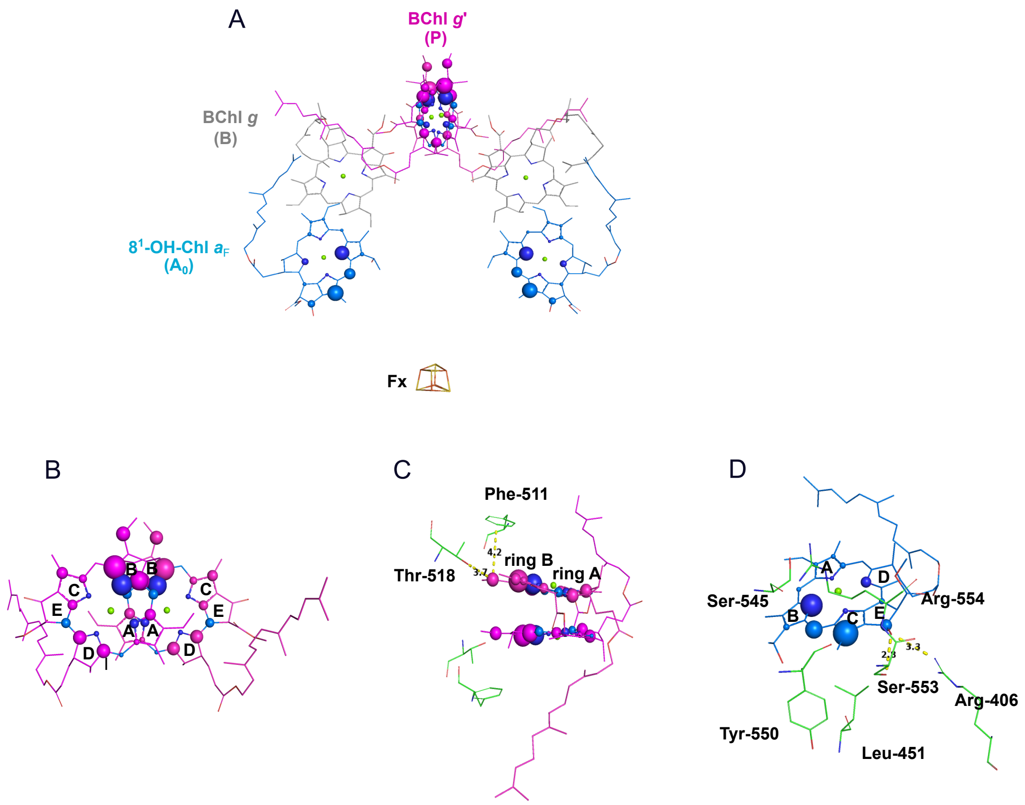

Figure S6. Map of local electron-spin densities for ^{13}C and ^{15}N in the donor triplet state and radical anion state within the HbRC (**A**) based on solid-state photo-CIDNP intensities. Positive ^{13}C signals are depicted in red, while negative ones appear in blue; negative ^{15}N signals are marked in dark blue. The size of the spheres roughly corresponds to the signal intensity. Spin density patterns of the overlapped special pair in the donor triplet state (**B**), and demonstrating interactions with nearby amino acid residues around ring B (**C**). The distances are represented by dotted lines and labels. The spin density patterns of the primary electron acceptor in the radical pair anion state are depicted, highlighting interactions where Ser-553 and Arg-406 form a hydrogen bond with the 13^1-C keto oxygen. [The figure was prepared with the PyMOL Molecular Graphics System, version 2.5 Schrödinger, LLC].

Table S4. Chemical shifts for the aromatic region of active cofactors (donor and acceptor) observed photo-CIDNP MAS NMR experiment of HbRC, the RCs of *R. sphaeroides* WT and PSII of spinach (unit: ppm of chemical shift scale)

	HbRC*			*R. sphaeroides* WT					PSII of spinach		
	Donor	Acceptor	OCSD $\Delta\delta$ (Acc.-Don.)	Donor			Acceptor	OCSD $\Delta\delta$ (Acc.-Don.)	Donor	Acceptor	OCSD $\Delta\delta$ (Acc.-Don.)
IUPAC no.	BChl *g'*	8^1-OH-Chl a_F,		BChl *a* (P_L), [3,5]	BChl *a* (P_M), [3,5]	Averaged (P_L+P_M)	BPheo *a*, [4,5]		Chl *a* [6]	Phe *a* [6,7]	
C1	155.2	157.3	2.1	144.1	144.8	144.45	141.2	-3.3	156	141.0	-15.0
C2	133.6	136.8	3.2	135.1	137.1	136.1	138.7	2.6	136.5	131.0	-5.5
C3	139.2	140.3	1.1	129.5	129.8	129.65	133.5	3.9	137.4	136.5	-0.9
C4	148.8	149.9	1.1	137.0	145.9	141.45	137.8	-3.6	150.1	137.0	-13.1
C5	97.2	96.2	-1.0	97.8	101.7	99.75	98.5	-1.3	98.1	97.0	-1.1
C6	166.1	153.8	-12.3	167.7	167.3	167.5	165.1	-2.4	154.3	156.0	1.1
C9	147.6	145.5	-2.1	160.8	159.8	160.3	162.7	2.4	146.0	148.5	2.5
C10	101.3	112.6	11.3	99.5	100.2	99.85	102.5	2.65	108.2	104.7	-3.5
C11	146.2	147.0	0.8	153.7	154.7	154.2	139.5	-14.7	151.6	138.0	-13.6
C12	120.7	134.6	13.9	120.3	128.8	124.55	126.9	2.4	133.7	128.95	-4.75
C13	127.9	127.3	-0.6	131.6	130.7	131.15	127.5	-3.7	129.5	130.0	0.5
C14	162.3	163.0	0.7	158.7	161.5	160.1	150.0	-10.1	162.2	151.0	-11.2
C15	108.9	102.6	-6.3	104.7	107.4	106.05	109.5	3.5	102.8	106.9	4.1
C16	151.0	157.8	6.8	149.0	158.4	153.7	157.7	4.0	157.4	160.7	3.3
C19	169.6	172.0	2.4	161.7	169.5	165.6	170.8	5.2	166.8	172.2	5.4
C20	92.0	93.2	1.2	95.4	104	99.7	94.2	-5.5	93.3	93.0	-0.3
SUM	2167.6	2189.9	22.3	2146.6	2201.6	2174.1	2156.1	-18.0	2183.9	2132.45	-51.45

*The chemical shifts of cofactors of HbRC were based on experimental data obtained from this study.

References

1. Qiu, N.W.; Jiang, D.C.; Wang, X.S.; Wang, B.S.; Zhou, F. Advances in the Members and Biosynthesis of Chlorophyll Family. *Photosynthetica* **2019**, *57*, 974–984, doi:10.32615/ps.2019.116.
2. Tsukatani, Y.; Yamamoto, H.; Mizoguchi, T.; Fujita, Y.; Tamiaki, H. Completion of Biosynthetic Pathways for Bacteriochlorophyll g in *Heliobacterium Modesticaldum*: The C8-Ethylidene Group Formation. *Biochim. Biophys. Acta - Bioenerg.* **2013**, *1827*, 1200–1204, doi:10.1016/j.bbabio.2013.06.007.
3. Sai Sankar Gupta, K.B.; Daviso, E.; Jeschke, G.; Alia, A.; Ernst, M.; Matysik, J. Spectral Editing through Laser-Flash Excitation in Two-Dimensional Photo-CIDNP MAS NMR Experiments. *J. Magn. Reson.* **2014**, *246*, 9–17, doi:10.1016/j.jmr.2014.06.007.
4. Sai Sankar Gupta, K.B.; Alia, A.; Buda, F.; De Groot, H.J.M.; Matysik, J. Bacteriopheophytin a in the Active Branch of the Reaction Center of *Rhodobacter Sphaeroides* Is Not Disturbed by the Protein Matrix as Shown by ^{13}C Photo-CIDNP MAS NMR. *J. Phys. Chem. B* **2013**, *117*, 3287–3297, doi:10.1021/jp3121319.
5. Gräsing, D. Photo-CIDNP MAS NMR — Generation, Transfer and Application of Nuclear Hyperpolarization, Ph.D. thesis, Universität Leipzig, Leipzig, Germany; 2019.
6. Janssen, G.J.; Bielytskyi, P.; Artiukhin, D.G.; Neugebauer, J.; de Groot, H.J.M.; Matysik, J.; Alia, A. Photochemically Induced Dynamic Nuclear Polarization NMR on Photosystem II: Donor Cofactor Observed in Entire Plant. *Sci. Rep.* **2018**, *8*, 17853, doi:10.1038/s41598-018-36074-z.
7. Egorova-Zachernyuk, T.A.; Van Rossum, B.; Boender, G.J.; Franken, E.; Ashurst, J.; Raap, J.; Gast, P.; Hoff, A.J.; Oschkinat, H.; De Groot, H.J.M. Characterization of Pheophytin Ground States in *Rhodobacter Sphaeroides* R26 Photosynthetic Reaction Centers from Multispin Pheophytin Enrichment and 2-D ^{13}C MAS NMR Dipolar Correlation Spectroscopy. *Biochemistry* **1997**, *36*, 7513–7519, doi:10.1021/bi962770m.
